# Treatment completion for latent tuberculosis infection in Norway: a prospective cohort study

**DOI:** 10.1186/s12879-018-3468-z

**Published:** 2018-11-19

**Authors:** Yvette Louise Schein, Tesfaye Madebo, Hilde Elise Andersen, Trude Margrete Arnesen, Anne Ma Dyrhol-Riise, Hallgeir Tveiten, Richard A. White, Brita Askeland Winje

**Affiliations:** 1 Perelman School of Medicine at the University of Pennsylvania, Philadelphia, PA USA; 2 Department of Pulmonary Medicine, Stavanger University Hospital, Stavanger, Norway; 3 Department of Pulmonary Medicine, TB unit, Stavanger University Hospital, Stavanger, Norway; 4 Department of Tuberculosis, Blood Borne and Sexually Transmitted Infections, Norwegian Institute of Public Health, Oslo, Norway; 5 Department of Infectious Diseases, Oslo University Hospital, Oslo, Norway; 6 Institute of Clinical Medicine, University of Oslo, Oslo, Norway; 7 Dep. of Clinical Science, University of Bergen, Oslo, Norway; 8 Department of Pulmonary Medicine, Oslo University Hospital, Oslo, Norway; 9 Department of Infectious Disease Epidemiology and Modelling, Norwegian Institute of Public Health, Oslo, Norway; 10 Department of Vaccine Preventable Diseases, Norwegian Institute of Public Health, Oslo, Norway

**Keywords:** Latent tuberculosis infection, Preventive treatment, Rifapentine, Isoniazid, Rifampicin, Chemoprophylaxis, Compliance, Screening, Surveillance

## Abstract

**Background:**

Successful treatment of latent tuberculosis infection (LTBI) is essential to reduce tuberculosis (TB) incidence rates in low-burden countries. This study measures treatment completion and determinants of non-completion of LTBI treatment in Norway in 2016.

**Methods:**

This prospective cohort study included all individuals notified with LTBI treatment to the Norwegian Surveillance System for Infectious Diseases (MSIS) in 2016. We obtained data from MSIS and from a standardized form that was sent to health care providers at the time of patient notification to MSIS. We determined completion rates. Pearson’s chi squared test was used to study associations between pairs of categorical variables and separate crude and multivariable logistic regression models were used to identify factors associated with treatment completion and adverse drug effects.

**Results:**

We obtained information on treatment completion from 719 of the 726 individuals notified for LTBI treatment in 2016. Overall, 91% completed treatment. Treatment completion was highest in the foreign-born group [foreign-born, *n* = 562 (92%) vs Norwegian-born, *n* = 115 (85%), *p* = 0.007]. Treatment completion did not differ significantly between prescribed regimens (*p* = 0.124). Adverse events were the most common reason for incomplete treatment. We found no significant differences in adverse events when comparing weekly rifapentine (3RPH) with three months daily isoniazid and rifampicin (3RH). However, there were significantly fewer adverse events with 3RPH compared to other regimens (*p* = 0.037). Age over 35 years was significantly associated with adverse events irrespective of regimen (*p* = 0.024), whereas immunosuppression was not significantly associated with adverse events after adjusting for other variables (*p* = 0.306). Treatment under direct observation had a significant effect on treatment completion for foreign-born (multivariate Wald *p*-value = 0.017), but not for Norwegian-born (multivariate Wald *p*-value = 0.408) individuals.

**Conclusions:**

We report a very high treatment completion rate, especially among individuals from countries with high TB incidence. The follow-up from tuberculosis-coordinators and the frequent use of directly observed treatment probably contributes to this. Few severe adverse events were reported, even with increased age and in individuals that are more susceptible. While these results are promising, issues of cost-effectiveness and targeting treatment to individuals at highest risk of TB are important components of public health impact.

**Electronic supplementary material:**

The online version of this article (10.1186/s12879-018-3468-z) contains supplementary material, which is available to authorized users.

## Background

Treatment of latent tuberculosis infection (LTBI) in groups at high risk for tuberculosis (TB) disease is a cornerstone in the global strategy to eliminate TB [1]. Norway has a mandatory screening program for TB and LTBI, which includes immigrants from high TB incidence countries, pre-employment screening, and outbreak management [2]. In addition, LTBI management is recommended prior to iatrogenic immunosuppression. In 2016, immigrants under the age of 35 from countries with TB incidence rates (IR) > 40 per 100,000 population (as estimated by WHO) were eligible for screening with IGRA (or equivalent) upon arrival in Norway. In addition, immigrants > 15 years of age were screened for TB with a chest X-ray. In Norway’s national guidelines, LTBI treatment is strongly recommended for children under the age of 5, contacts, those with fibrotic lesions on chest X-ray, or those with select immunosuppressive conditions (HIV-infection, haemodialysis, solid organ transplants, malignancies, or prior to iatrogenic immunosuppression). LTBI treatment is conditionally recommended for children aged 5–14 years, those with calcifications on chest X-ray, those who are underweight, and individuals with long-term steroid treatment, diabetes mellitus, or drug addiction. Being foreign-born was not considered a single criterion for priority for treatment at the time of the study [3].

The use of LTBI treatment has rapidly increased in Norway in recent years, stabilizing at around 750 cases per year [4, 5]. Individuals prescribed LTBI treatment are reported to the Norwegian Surveillance System for Infectious Diseases (MSIS) [6]. However, treatment completion, an important indicator of the impact, safety, and cost effectiveness of the screening program, is not routinely reported.

LTBI treatment completion rates vary considerably across risk-groups and settings [7], and long duration and adverse events are well-known barriers to treatment completion [7–12]. A Norwegian study from 2009 found overall high LTBI treatment completion, and no adverse events requiring hospital admission [13]. Since then, Norwegian guidelines have increasingly targeted groups at high risk for TB reactivation, including older individuals with more comorbidities and recent immigrants to Norway [3]. Recent immigrants are often difficult to follow-up due to their mobility and a lack of government-issued identification numbers [14, 15]. Additionally, the increasing use of immunosuppressants may pose new challenges to LTBI treatment strategy.

The most common LTBI regimen is a daily combination of rifampicin and isoniazid for three months (3RH). The increasing use of weekly-administered rifapentine (3RPH) [8], a newer and less-studied regimen, highlights the need for strengthened LTBI treatment surveillance. National guidelines currently recommend directly observed treatment (DOT) for this new 3RPH regimen.

Severe adverse events due to preventive treatment are primarily related to isoniazid induced peripheral neuropathy and hepatotoxicity, with higher rates found in those aged > 35 years [12, 16], and hepatotoxicity, gastrointestinal intolerance, and hypersensitivity from rifamycins [12]. We question whether tolerance to adverse effects may be context specific, with lower tolerance in a country where TB is rare. If significant scale-up of LTBI treatment is indicated, information about adverse events and treatment completion by regimen will be highly relevant.

In Norway, prescription of TB drugs, including LTBI treatment, is the responsibility of pulmonologists, infectious disease specialists, or paediatricians who work primarily in hospital settings. The role of “TB coordinator” was introduced in the national TB control programme in 2003 to strengthen patient support and coordinate TB control between all healthcare levels [2]. One key responsibility of the coordinator is to establish a treatment plan for every individual starting TB treatment. The plan clearly states the responsibilities of all partners involved and seeks to maximize patient-involvement.

The objective of this study is to measure LTBI treatment completion and determinants of non-completion in all individuals notified to MSIS in 2016. We also assess the risk of severe adverse events related to treatment, and their consequences for the LTBI treatment strategy, and explore the use of associated healthcare resources.

## Methods

### Study participants

This is a nationwide prospective cohort study, including all individuals reported with LTBI treatment to MSIS between January 1, 2016 and December 31, 2016. The protocol is available as supplementary material (Additional file 1:). Data included demographic and clinical information available through MSIS, and additional data on treatment completion, adverse events, patient support, and use of healthcare resources collected through a standardized treatment completion form that was sent to prescribing clinicians and TB coordinators at the time the individual was reported to MSIS (form with translation, see Additional file 2: a, b). If the form was not returned, we sent one reminder by mail before calling the prescribing clinician or responsible TB coordinator. If multiple forms were returned for the same individual, we verified conflicting data with a call to the responsible physician or TB coordinator. If we were unable to verify conflicting data, such as the number of visits with a physician or TB coordinator, we calculated the mean value of the reported data before inclusion in the analyses.

### Definitions

LTBI treatment completion was measured as reported by the responsible clinician, and verified by the duration of treatment (measured in days) for the different regimens. Information on adverse events was only reported if it led to interruption (treatment was stopped temporarily, but later continued) or termination (stopped completely) of treatment. Severity of hepatotoxicity was based on reported increase in the liver enzymes alanine aminotransferase (ALAT) and aspartate transaminase (ASAT) and bilirubin in blood samples. Severity was classified by upper limits of normal values (ULN) for serum levels of ALAT or ASAT, consistent with the Common Terminology Criteria for Adverse Events (CTCAE4) [17]: grade 1 (>ULN -3.0 x ULN), grade 2 (> 3.0–5.0 x ULN), grade 3 (> 5.0–20.0 x ULN), and grade 4 (> 20.0 x ULN). Other blood test results were not routinely collected. Remaining adverse events were classified by their clinical symptoms.

### Statistical analysis

We used STATA 2015 for statistical analysis. We present descriptive statistics for continuous variables as means and standard deviations for symmetrical data, and medians and interquartile range (IQR) for skewed data. Pearson’s chi squared or Fisher’s exact test, as appropriate, assessed associations between pairs of variables.

Additionally, we ran two separate logistic regression models. The first model analysed the association between treatment non-completion and origin (Norwegian-born/foreign-born), age (>/< 35 years), gender, and type of treatment support (self-administered treatment/daily DOT/weekly DOT). Treatment regimen could not be included in the model because of its association with DOT. Similarly, we could not include both establishment of treatment plan and use of DOT in the model because the two are related. The final model did not include either the establishment of a treatment plan or immunosuppression because these were not found to be significantly associated with treatment completion in crude analyses.

To investigate if the association between treatment non-completion and treatment support was modified by age, sex, or origin we ran separate models. We ran the baseline model (treatment non-completion = treatment support + origin + age + sex) and then subsequently tested for interaction between treatment support and age, sex, or origin, i.e. (treatment support*age)/ (treatment support*sex)/ (treatment support*origin). We then performed likelihood ratio tests comparing the separate models to the baseline model. We found no statistically significant effect of age or sex on treatment support. We found a significant effect of origin on treatment support and therefore stratified the effect of treatment support on treatment non-completion by origin in the model. We also tested for an interaction between origin and age and origin and sex. No such interactions were identified.

Information on age and origin was complete. Information on treatment completion was missing for six individuals, and treatment support was missing for 13. Since missing information is unlikely random, we used multiple imputations to investigate the effect of missing data on the model. We imputed five datasets using chained equations (“mi impute chained” command) and recreated the complete-case analyses using the imputed data.

The second model analysed the association between interruption or termination of treatment because of adverse events (yes/no), and treatment regimens (3HR/3RPH/Other), age (>/< 35 years), and immunosuppression (yes/no). One individual had missing information for treatment regimen, and for this person we coded treatment regimen as “other”.

To investigate if the association between adverse events and treatment regimen was modified by age or immunosuppression, we ran two models. We ran the baseline model (adverse effect = treatment regimens + age + immunosuppression) and subsequently tested for interaction between treatment regimens and age or immunosuppression, i.e. (treatment regimens*age)/ (treatment regimens*immunosuppression). We also ran a model investigating whether there was an interaction between age and immunosuppression, i.e. (age*immunosuppression). We then performed likelihood ratio tests comparing the separate models to the baseline model. We found no statistically significant effect of age or immunosuppression on treatment regimens. We could not test the interaction between age and immunosuppression due to the small number of cases.

## Results

### Study population

In 2016, MSIS received 747 notifications of the prescription of LTBI treatment. We later excluded 21 cases because the treatment was completed in 2015 (delay in reporting), which left 726 individuals for follow-up in the prospective cohort. Table 1 presents baseline characteristics of the study population by origin.

**Table 1:** Baseline characteristics of the study population by origin, *n* = 726

Baseline Characteristics	Norwegian-born	Foreign-born	Total	p-value
Number of individuals	131 (100)	595 (100)	726 (100)	
Gender				0.115
Male	69 (53)	358 (60)	427 (59)	
Age at notification				> 0.001
< 5 years	37 (28)	25 (4)	62 (9)	
5–14 years	10 (8)	132 (22)	142 (20)	
15–34 years	33 (25)	315 (53)	348 (48)	
35–64 years	36 (27)	115 (19)	151 (21)	
> 65 years	15 (11)	8 (1)	23 (3)	
Risk groups^a^				
Any risk factor^a^	121(92)	342 (58)	463 (64)	
Recent exposure, contacts	68 (52)	95 (16)	163 (22)	> 0.001
Contacts < 5 years	35 (27)	3 (0.5)	38 (5)	
Positive chest X-ray, any	7 (5)	60 (10)	67 (9)	0.090
Fibrotic lesions	2 (2)	17 (3)	19 (3)	0.388
Immunosuppressive condition	51 (39)	72 (12)	123 (17)	> 0.001
HIV infection	–	12 (2)	12 (2)	
Chronic renal disease	1 (1)	4 (1)	5 (1)	
Diabetes, any	1 (1)	4 (1)	5 (1)	
Underlying disease relevant for Immunosuppressive treatment^b^	13 (10)	10 (2)	23 (3)	
Other medical conditions^c^	36 (7)	42 (7)	78 (15)	
Interferon Gamma Release Assay (IGRA)				> 0.001
Positive	94 (72)	577 (97)	671 (92)	
Negative	15 (11)	5 (1)	20 (3)	
Inconclusive	2 (2)	–	2 (0)	
Missing or unknown	20 (15)	13 (2)	33 (5)	
Regimen				> 0.001
3RH daily^I^	97 (74)	302 (51)	399 (55)	
3RPH weekly^II^	30 (23)	276 (46)	306 (42)	
4R or 6H monotherapy daily^III^	3 (2)	15 (3)	18 (2)	
Other^IV^	–	2 (0)	2 (0)	
Missing	1 (1)	–	1 (0)	
TB IR (per 100,000) in country of birth^**#**^				
< 150	na	267 (45)	267 (45)	
150–200	na	127 (21)	127 (21)	
> 200	na	197 (33)	197 (33)	
Missing	na	4 (1)	4 (1)	

Data are presented as n (%) or median [interquartile range]. % refers to columns and not rows

^a^According to Norwegian guidelines for the management and control of tuberculosis: with strong or conditional recommendation for LTBI treatment (age < 15 years, known exposure, positive chest X-ray or immunosuppressive condition)

^b^Includes rheumatologic-, dermatologic-, neurologic- and gastroenterological medical conditions

^c^Other medical conditions include unspecified immunosuppressive conditions reported by the clinician

^I^3RH: rifampicin (R) and isoniazid (H) daily for three months

^II^3RPH: rifapentine (RP) and isoniazid (H) in 12 weekly doses

^III^Rifampicin (R) monotherapy daily for four months or isoniazid (H) monotherapy daily for six months

^IV^Others include full-course TB treatments for two individuals for two and four months respectively

# Four immigrants had missing information on country of birth

Eighteen percent of the individuals (*n* = 131) were Norwegian-born and 82% (*n* = 595) were foreign-born. Information on country of birth was missing for four individuals. Among foreign-born, 197 (33%) were born in a country with a WHO estimated [18] TB incidence rate (IR) of > 200 per 100,000 population, Table 1. Forty-three percent of immigrants (*n* = 253) had no additional risk factors for TB progression other than being foreign-born. Of these, 94 (35%) arrived from countries with TB IR < 150, 66 (52%) with IR 150–200, and 91 (46%) with IR > 200 per 100,000 population (2 missing). In total, 314 (53%) of the immigrants had lived in Norway for less than one year at the time of notification, and were therefore classified as recent immigrants.

The Norwegian-born group was more likely to be part of contact tracing, have at least one medical risk factor, and have a negative interferon-gamma release assay result (IGRA; QuantiFERON TB-Gold (QFT®)) prior to treatment onset, Table 1. Age distribution differed between Norwegian and foreign-born, with a higher proportion of either young or old patients in the Norwegian-born group. 3RH (55%) was the most common regimen, although the use of the newest regimen, 3RPH (42%), was frequent, and its use increased over the study period. The 3RPH regimen was more commonly prescribed among foreign-born (46%) compared to Norwegian-born (23%).

### Treatment completion

Information on treatment completion was obtained for 719 (99%) of the individuals who started LTBI treatment. Table 2 presents treatment completion and the reasons for not completing treatment. Overall, 91% completed their treatment course as reported by the prescribing physician.

**Table 2:** Treatment completion and reasons for non-completion by treatment regimen, Norway 2016

Treatment completion	3RH^I^ daily	3RPH^II^ weekly	Other^III^	Total
Number of individuals	399	306	21	726
Duration of treatment (days), median [IQR]*	90 [73–156]	77 [70–98]	–	–
Treatment completion				
Completed according to physician^a^	357 (89.5)	284 (92.8)	17 (80.9)	658 (90.6)
Missing information	3 (0.8)	4 (1.3)	–	7 (1.0)
Incomplete treatment	39 (10)	18 (5.9)	4 (19.0)	61 (8.4)
Reasons for incomplete treatment				
LTBI excluded	4 (1.0)	–	–	4 (0.6)
Diagnosed with TB disease	1 (0.3)	–		1 (0.1)
Patient choice	4 (1.0)	2 (0.7)	–	6 (0.8)
Lost to follow-up	1 (0.3)	3 (1.0)	–	4 (0.6)
Other or unknown	3 (0.8)	2 (0.7)		5 (0.7)
Termination due to adverse effects^b^	26 (6.5)	11 (3.4)	4 (19.0)	41 (5.6)
Hepatotoxicity (grade 1–2)^c^	8 (2.0)	–	–	8 (1.1)
Hepatotoxicity (grade 3–4)^c^	5 (1.6)	2 (0.7)	1 (4.8)	8 (1.1)
Gastrointestinal symptoms	10 (2.5)	8 (2.6)	2 (9.5)	20 (2.8)
Fatigue	6 (1.5)	8 (2.6)	1 (4.8)	15 (2.1)
Flu-like symptoms	2 (0.5)	4 (1.3)	1 (4.8)	7 (1.0)
Skin rash	2 (0.5)	1 (0.3)	1 (4.8)	4 (0.6)
Peripheral neuropathy	1 (0.3)			1 (0.1)
Joint pain	–	2 (0.6)	–	2 (0.3)
Other symptoms^d^	2 (0.5)	3 (1.0)	2 (9.5)	7 (1.0)

Data are presented as n (%) or median [interquartile range]

^I^3RH: rifampicin (R) and isoniazid (H) daily for three months

^II^3RPH: rifapentine (RP) and isoniazid (H) in 12 weekly doses

^III^Other: rifampicin (R) monotherapy daily for four months (*n* = 5), Isoniazid (H) monotherapy for six months (*n* = 13) or combination therapy for TB disease (*n* = 2) and missing information about drug regimen (n = 1)

*Duration of treatment for those where the clinician reported the treatment as completed

^a^The responsible clinician reported that the planned treatment was completed

^b^Many reported more than one adverse effect

^c^Severity of hepatotoxicity was classified according to Common Terminology Criteria for Adverse Events (CTCAE), ULN = upper limits of normal value for serum levels of liver function, grade 1 (>ULN -3.0 × ULN), grade 2 (> 3.0–5.0 × ULN), grade 3 (> 5.0–20.0 × ULN), and grade 4 (> 20.0 × ULN)

^d^Includes headache, sleep disorder, and unstable international normalized ratio (INR) for prothrombin time

Treatment completion did not differ significantly for different regimens (*p* = 0.124). Foreign-born individuals were more likely to complete treatment compared to Norwegian-born [foreign-born, *n* = 562 (92%) vs Norwegian-born, *n* = 115 (85%), *p* = 0.007]. They were also more likely to be prescribed with 3RPH [foreign-born, *n* = 276 (46%) vs Norwegian-born *n* = 30 (23%) *p*-value 0.001], a regimen for which DOT is recommended. Foreign-born individuals were also more likely to be treated under DOT, even for regimens for which DOT is not routinely recommended [foreign-born, *n* = 151 (47%) vs Norwegian-born *n* = 28 (28%) *p* = 0.002]. Among recent immigrants, 294 (94%) completed their treatment, 48% were prescribed with 3RPH, and 65% were treated under DOT while on regimens other than 3RPH.

In crude logistic regression models, origin, age, sex, and treatment support were significantly associated with treatment non-completion. In a multivariable logistic regression model, we found that neither origin, age over 35 years, or sex were significantly associated with treatment non-completion. However, the effect of treatment support was modified by origin, with significantly lower risk of non-completion with daily and weekly DOT compared to self-administration in the foreign-born group. Treatment support had no significant effect on treatment completion in the Norwegian-born group, Table 3.

**Table 3:** Associations between the outcome of treatment non-completion and the variables treatment support, origin, age and sex (*n* = 726)

Covariates	Univariable			Multivariable			
	n	c OR	p	a OR	SE	p	95% CI
Origin							
Foreign-born	595	1 (ref)		1(ref)			
Norwegian-born	131	2.1	0.016	0.8	0.342	0.602	0.34–1.84
Age group							
< 35	561	1 (ref)		1 (ref)			
> 35 years	165	1.9	0.026	1.7	0.521	0.093	0.92–3.09
Sex							
Female	299	1 (ref)		1 (ref)			
Male	427	0.5	0.017	0.6	0.093	0.106	0.36–1.10
Treatment support. Model with interaction term for origin: LRT p-value of interaction term = 0.049							
Effect of treatment support on treatment completion in foreign-born (multivariate wald p-value 0.017)							
Self-administered^a^	174	1 (ref)		1 (ref)			
DOT daily^b^	151	0.2	0.005	0.3	0.150	0.017	0.10–0.80
DOT weekly^c^	261	0.4	0.009	0.4	0.162	0.027	0.22–0.91
Effect of treatment support on treatment completion in Norwegian-born (multivariate wald p-value 0.468)							
Self-administered^a^	80	1 (ref)		1 (ref)			
DOT daily^b^	28	1.7	0.374	2.1	1.332	0.229	0.62–7.26
DOT weekly^c^	19	0.9	0.928	1.0	0.868	0.961	0.20–5.34

*OR* Odds ratio, *SE* standard error, *DOT* Direct Observed Treatment for part of or the full treatment period

^a^Self-administered include those who managed their treatment themselves or were given weekly pill boxes

^b^daily DOT: those who were administered daily treatment under direct observation

^c^weekly DOT: those who were administered weekly rifapentine and isoniazid under direct observation

We ran a sensitivity analysis by rerunning the analyses in Table 3 with a multiple imputed dataset (using chained equations). The sensitivity analysis showed no impact on the overall results, and the results are available as supplementary material (Additional file 3).

### Adverse events

Adverse events was the most common reason reported for incomplete treatment in all groups. The total number of adverse events according to the study definitions was 47 (6.5%). In total, 41 (5.6%) individuals terminated treatment, Table 2, and an additional six (0.8%) individuals interrupted treatment to control adverse events but later continued and completed treatment. These six individuals are recorded as complete treatment in Table 2. Three of these temporary interruptions (3RH) were due to hepatotoxicity (one grade 2 and two grade 3), two were due to gastrointestinal symptoms, (3RPH and H) and one to fatigue (3RPH). The duration of the interruption was 2, 4, 7, 22, 28 and 113 days respectively.

In a multivariable logistic regression model, we found that regimen was borderline associated with adverse events (*p*-value = 0.065). There were no significant differences when comparing 3RH vs 3RPH, nor 3RH vs Other (R or H monotherapy or full course TB treatment). However, there were significantly fewer adverse events with 3RPH compared to "other" regimens (*p* = 0.037). Age over 35 years was significantly associated with having more adverse events, even after adjusting for regimen, whereas immunosuppression was not significantly associated with having adverse events after adjusting for other variables, Table 4. No deaths were reported.

**Table 4:** Associations between adverse events^a^, regimen, age, and immunosuppression, n = 726

Covariates		Univariable		Multivariable			
	n	c OR	p	a OR	SE	p	95% CI
Regimen (Wald test for overall variable in adjustment model, *p* = 0.065)							
3RH^b^	399	1 (ref)		1 (ref)			
3RPH^c^	306	0.57	0.097	0.60	0.209	0.141	0.30–1.18
Other^d^	21	3.99	0.012	2.37	1.360	0.131	0.78–7.30
Age							
< 35 yrs	561	1 (ref)		1 (ref)			
> 35 yrs	165	3.62	< 0.001	2.51	2.25	0.024	1.13–5.60
Immunosuppression							
No immunosuppression	603	1 (ref)		1 (ref)			
Immunosuppression, any	123	3.39	< 0.001	1.55	0.66	0.306	0.67–3.57

*OR* Odds ratio, *SE* standard error

^a^Adverse effects leading to termination or interruption of treatment, *n* = 47

^b^3HR, 3 months daily rifampicin and isoniazid

^c^3RPH, 12 weekly doses of rifapentine and isoniazid

^d^Other; rifampicin monotherapy (*n* = 5), isoniazid monotherapy (*n* = 13), combination therapy for TB disease (*n* = 2) and 1 missing information

Duration of treatment prior to termination ranged from 2 to 122 days, with a median of 25 days (IQR 6–84). For the two 3-month regimens combined (3RH +3RPH), 61% of those who terminated treatment completed less than half of the prescribed regimen.

Among those who met the study-definition for adverse effects, 22 (47%) had one symptom, 16 (34%) had two, and nine (19%) had three. There was no significant difference in number of symptoms reported between those who interrupted and those who terminated treatment (*p* = 0677). Gastrointestinal symptoms was the most common complaint in both age groups, followed by hepatotoxicity and fatigue, Table 2. Two individuals were classified with grade 4 hepatotoxicity. One was a child who was diagnosed with toxic hepatitis after two weeks of LTBI treatment. Treatment was immediately stopped, allowing transaminase values to normalize, and the patient fully recovered. The other was a middle-aged individual who developed hepatotoxicity with high transaminase values after eight weeks of LTBI treatment. The treatment was immediately stopped, and transaminase values normalized after six weeks. Both individuals received 3RH regimen and were managed as outpatients. Additionally, eight individuals were classified as having hepatotoxicity grade 3. Two of these were hospitalized due to complex comorbidities. The remaining were treated as outpatients, and transaminase values normalized after treatment was stopped. The 3RH regimen was prescribed in 16 out of 19 (84%) patients who interrupted or terminated treatment due to hepatotoxicity.

### Patient support, drug administration, and use of healthcare resources

A treatment plan was established prior to treatment for 655 (90%) individuals. Foreign-born individuals were more likely to have a treatment plan compared with Norwegian-born individuals (*n* = 544, 91% vs *n* = 111, 85%, p 0.017).

DOT, either for part of or the whole treatment period, was reported in 173 (43%) of individuals prescribed with 3RH, in 280 (92%) with 3RPH, and in six (29%) with “other”, Table 5. Home-visiting nurses provided DOT in 324 (71%) cases, the family in 11 (2%), and outpatient hospital clinics, assisted living facilities, refugee centres, work places, or general practitioners in 57 (12%). Information about the DOT provider was missing for 65 (15%) individuals.

**Table 5:** Drug-administration by regimen, n = 726

Drug administration	3RH^a^ daily	3RPH^b^ weekly	Other^c^	Total
Number of individuals	399 (100)	306 (100)	21 (100)	726 (100)
Self-administered	220 (55)	20 (7)	14 (67)	254 (35)
Direct observed treatment				
Whole period	132 (33)	269 (88)	3 (14)	404 (56)
Part of period	41 (10)	11 (4)	3 (14)	55 (8)
Missing information	6 (2)	6 (2)	1 (5)	13 (2)

Data are presented as n (%)

^a^rifampicin (R) and isoniazid (H) daily for three months

^b^rifapentine (RP) and isoniazid (H) in 12 weekly doses

^cI^rifampicin (R) monotherapy daily for four months (n = 5), Isoniazid (H) monotherapy for six months (n = 13) or combination therapy for TB disease (n = 2) and missing information (n = 1)

^d^Other included: hospital outpatient clinic, assisted living facilities, refugee centres, work-places or with general practitioners.

The median number of consultations with a medical doctor was 1.5 (IQR 1–8, range 1–11), and 1.5 (IQR 0–7, range 0–14) with nurse. Almost half (48%) had only one consultation with a doctor. We found no significant difference in the mean number of consultations with a doctor or nurse between foreign-born and Norwegian-born individuals. Among the 658 individuals who completed treatment, 397 (60%) had their last consultation in the hospital before (*n* = 334, 51%), or at the time of treatment initiation (*n* = 63, 9%). Only 193 (29%) had a consultation in the hospital more than six weeks after the treatment start date.

## Discussion

This one-year prospective study found a 91% LTBI treatment completion rate in Norway. Treatment completion did not differ significantly by regimen. Completion was higher among foreign-born than Norwegian-born individuals. Treatment under DOT had a significant effect on treatment completion for foreign-born individuals, but not in the Norwegian-born group. The primary reason for not completing treatment was adverse events. The majority of these were mild to moderate in severity, although medically significant (grade 3) and severe (grade 4) hepatotoxicity led to termination of treatment in 1.1% of participants. Few individuals were lost to follow-up. Individual treatment plans were established for the majority, and DOT was common, even with regimens where this is not routinely recommended.

### Treatment completion

The treatment completion rate of 91% is consistent with the 89% previously reported in Norway [13]. Direct comparison with previous studies is difficult due to differences in study designs, regimens, and the populations under study. Completion rates are commonly reported as inversely related to treatment length [7]. Unsupervised six to nine months of isoniazid treatments in the US have shown around 50% completion rates [10, 11, 19, 20], 3–4 months RH regimens are reported at 85–90% [13, 21–23] and recent studies report similarly high completion rates with weekly (supervised) 3RPH administration [24–28]. Our results are consistent with a 2017 review [29] reporting no important differences in efficacy and completion rates for the 3RPH regimen when compared to other regimens. The review, however, did support a higher likelihood of completion with a shorter regimen. Additionally, lower loss to follow up has been associated with immunocompromised patients, being part of a contact investigation, and the shorter, rifamycin-based regimens [30].

Our high completion rate is surprising given that the majority of patients were recent arrivals to Norway and were born in countries with a high TB prevalence, factors often associated with poor access and adherence to health services. A Swedish study reported 76% LTBI treatment completion over a six year period (2002–2007) overall, but only 68% completion among recent immigrants, (< 1 years residence) with immigrants of Somali origin having the lowest completion rates [23]. Similarly in Japan, a study reported significantly higher completion rates in Japanese versus foreign-born individuals [31]. In our setting, the most recent immigrants completed treatment at a higher rate than other immigrants, and immigrants as a whole completed treatment to a higher extent than Norwegian-born individuals. This finding is unexpected because immigrants in Norway tend to under-utilize other preventive health services, such as mammography [32]. Foreign-born individuals in this study were more likely to have a treatment plan established prior to LTBI treatment, be treated under direct observation, and were more commonly prescribed the 3RPH weekly regimen compared to Norwegian-born individuals. These findings may explain such high rates of completion among recent immigrants. Treatment support, which was coded separately for daily or weekly DOT to reflect treatment regimens, was significantly associated with treatment completion among foreign-born individuals when controlling for other co-variates. However, we were unable to control for adverse effects as a co-variate in the analyses, since this information was only recorded when not completing treatment.

Another explanation may be the role of TB coordinators, mostly specialized nurses, which is unique in a northern European setting. The TB coordinators are actively involved in implementation of TB control activities, both on individual and system levels. They know their patients well, and this may mitigate loss to follow-up. Previous studies have shown that social interventions such as adherence coaching, contingency contracting, enhanced outreach, and home visits improve treatment completion and may benefit patients who are at risk of progressing to active TB [33, 34]. In Norway, such activities are initiated by TB coordinators.

### Adverse events

Adverse events was the most common reason for not completing treatment in both the Norwegian and foreign-born groups. This is consistent with other studies [35]. The proportion of individuals who discontinued LTBI treatment due to side-effects (5.6%) in this study is similar to the 7.6% reported in the Netherlands [21], 6.4 and 5.9% reported in the USA [35], and 7% reported in Norway in 2009 [13], but lower than the 12% reported from Australia [36]. Similarly, a recent study in Taiwan that compared treatment completion and cost effectiveness for 3RPH versus H reported higher completion in the 3RPH regimen due to the shorter treatment period and reduced likelihood of severe side effects [37]. This is also consistent with our results.

The severity of reported adverse events varied. Since the treatment is preventive rather than curative, mild to moderate side effects may influence both the patient’s and the doctor’s motivation to continue treatment. The rate of clinically significant and severe hepatotoxicity (grade 3 and 4) reported in this study (1.1%) is lower than a median rate of 1.8% (range 0.1–11.9%) reported in a study on age-related hepatotoxicity following 6-9 months of isoniazid [16]. In accordance with other studies, we found a higher rate of grade 3 and 4 hepatotoxicity with increasing age [7, 16, 36], lower rates with 3RPH compared with other regimens [20, 26, 35, 38], rare need for hospitalization, and no deaths [13, 16, 27, 36, 39, 40].

### Patient support and use of health care resources

Norway’s high completion rate must also be seen in relation to the resources used to achieve it. Two elements that make preventive treatment in Norway relatively costlier than elsewhere are the frequent use of DOT and the highly specialized care levels. Almost two thirds of the patients involved in the study received treatment under DOT. Norwegian guidelines do not routinely recommend DOT for treatment regimens other than 3RPH. The increasing use of this 12-dose regimen (3RPH), with weekly administration rather than a prolonged daily regimen, will reduce DOT associated costs. Further, a recent randomized clinical trial found that there was not a significant difference in completion rates for directly observed 3RPH versus self-administered 3RPH treatment, which may change current recommendations for supervised 3RPH treatment [41].

Providing preventive treatment through subspecialist physicians rather than primary care is costly. However, training a larger group of primary care doctors about the details of TB and its prevention and treatment in low incidence settings such as Norway would require substantial effort. The greatest cost, as well as the greatest benefit, may be attributable to having dedicated TB coordinators. In the current study, 90% of patients had treatment plans established by TB coordinators.

### Strengths and limitations

The strengths of the study include the population-wide approach, the comprehensive information available for every individual, the standardized classification of severity of hepatotoxicity, and our very low rate of loss to follow-up. The sensitivity of our reporting system for LTBI treatment is assumed to be high since information on TB-related prescriptions is collected independently, and missing data is routinely monitored to ensure complete information.

Limitations include that completion rates and adverse events for the various regimens may reflect systematic differences in treatment assignment and clinician assessment rather than differences in the regimens themselves. We have, when possible, adjusted for this in our analyses. Judgment of treatment completion was made by the treating physician, often based on only one consultation at the beginning of the treatment course, in addition to information received from the TB coordinator and the DOT-provider. The question about completion was, however, followed up with a statement of the number of days on treatment and overseen by the TB coordinator. Adverse drug events that did not lead to interruption or termination of treatment were not recorded. Therefore, we could not control for this as a co-variate. We may have also missed some adverse effects and mild degrees of toxicity.

## Conclusions

This study indicates that with well-organized health services, high LTBI treatment completion is possible, even in high-risk immigrant groups. We report high treatment completion across all subgroups. Treatment completion is the end-point of Norway’s LTBI screening strategy. Knowledge of the treatment completion rate and adverse events are crucial in the assessment of the screening program’s impact and cost effectiveness. Although adverse events were the primary determinants of treatment non-completion, our results seem reassuring, even for more elderly and immunocompromised individuals, given adequate monitoring and follow-up during treatment. While this study has shown that DOT is very effective in ensuring treatment completion among hard to reach populations, further research is necessary to evaluate best practices from a cost-effectiveness standpoint.

## Additional files

Additional file 1: protocol. (PDF 376 kb)

Additional file 2: a, form sent to clinicians for information about treatment completion. b, translation of form sent to clinicians for information about treatment completion. (ZIP 680 kb)

Additional file 3: a sensitivity analysis exploring the associations between treatment non-completion, and treatment support, origin, age and sex after imputation for missing values. (PDF 472 kb)

## Abbreviations

3RH: Daily Rifampicin and Isoniazid for 3 Months
3RPH: Weekly Rifapentine and Isoniazid for 3 months
ALAT: Alanine aminotransferase
ASAT: Aspartate aminotransferase
CTCAE: Common Terminology Criteria for Adverse Events
DOT: Directly Observed Treatment
H: Daily Isoniazid for 6 months
IGRA: Interferon-gamma release assay
IR: Incidence Rate
LTBI: Latent Tuberculosis Infection
MSIS: Norwegian Surveillance System for Infectious Diseases
NIPH: Norwegian Institute of Public Health
R: Daily Rifampicin for 4 months
TB: Tuberculosis
ULN: Upper Limits of Normal values
WHO: World Health Organization
