# Supplementary material for: Treatment completion for latent tuberculosis infection in Norway: a prospective cohort study
Source: BMC Infect Dis. 2018 Nov 19;18:587. doi: 10.1186/s12879-018-3468-z (PMC6245849; doi:10.1186/s12879-018-3468-z)
Supplement: Supplementary file 1 — protocol. (PDF 376 kb) [file 12879_2018_3468_MOESM1_ESM.pdf]

Outcome of treatment of latent tuberculosis infection in Norway; a prospective cohort study

Latent tuberculosis and preventive treatment

Treatment of latent tuberculosis infection (LTBI) in groups at high risk for tuberculosis (TB) disease is a cornerstone in the global strategy towards TB elimination.[1] The strategy has been further enhanced in the recently published WHO guidelines for the management of LTBI in low- and middle incidence countries.[2] Successful implementation of the LTBI treatment strategy is challenged by difficulties in identifying individuals at highest risk of reactivation, imperfect diagnostic tests, poor treatment adherence, risk of serious fatal drug adverse effects and high costs. Currently available treatment regimens have shown efficacy ranging from 60% - 90%.[2, 3] The potential benefit of treatment needs to be carefully balanced against the risk of drug-related adverse events.[2]

Norway has a mandatory screening program for TB, including newly arrived immigrants from high TB incidence countries, occupational screening and outbreak management.[4] The use of LTBI treatment has rapidly increased in Norway in recent years and has stabilized around 750 cases per year.[5] Most cases are treated with a combination of rifampicin and isoniazid for three months.

Cases prescribed LTBI treatment are notified to the Norwegian Surveillance System for Communicable Diseases (MSIS).[6] Treatment outcome is not routinely reported. A study from 2009 found overall high LTBI completion rates and no adverse events requiring hospital admission.[7] Since then, Norwegian guidelines have increasingly targeted groups at high risk for reactivation. Groups receiving LTBI treatment today may therefore have higher age and higher rate of comorbidities. Rifapentine for weekly administration is expected to become available soon, adding on to the need for strengthened surveillance. Current Norwegian Guidelines for LTBI management only includes routine follow-up visit by the clinician after 6 weeks if there is a specific concern. The study may also add information to whether consultations at end of treatment should be recommended.

The risk of severe side effects from preventive treatment is mainly related to isoniazid induced hepatotoxicity with higher rates in those aged ≥35 years [8], and hepatotoxicity, peripheral neuropathy and hypersensitivity from rifamycins.[9] We have limited information on drug side effects and to what extent this causes treatment interruption or termination in the current Norwegian setting, and whether this differs among risk groups. Biological disease modifying anti-rheumatic drugs (bDMARDs) increases the risk of tuberculosis in people with LTBI. The increasing use of bDMARDs, also in older age groups, may pose new challenges to the LTBI treatment strategy. This population group has not been included in many of the randomized studies[10-12]. Also, we question whether tolerance to side effects may be context specific with lower tolerance in a country where TB is rare.

Follow-up of screening and treatment completion in newly arrived immigrants is a specific challenge, due to their high mobility and the lack of unique person-identifiable information.[13, 14] In Sweden younger patients, patients with Somali origin and asylum seekers were more likely to be non-completers.[15]

Rationale for Research:

Information about treatment completion is an important indicator to estimate potential impact of overall LTBI activities and also to measure its safety and cost-effectiveness. It reflects the TB control programs capacity to support adherence to treatment. Further, information about determinants of non-completion may provide knowledge for targeted interventions to risk-groups. This study is one of several ongoing projects initiated to provide input to a broader assessment of our national LTBI screening program. If significant scale-up of LTBI is to be recommended the information about side-effects and adherence is highly relevant.

Objective:

We aim to measure LTBI treatment completion and determinants of treatment non-completion in all cases notified to the Norwegian Surveillance System for Communicable Diseases (MSIS) over a twelve months period. Information from hospital pharmacies on prescription of LTBI treatment will be used to ascertain high notification coverage of LTBI treatment to MSIS. We will also measure drug adverse effects and its consequences for the LTBI strategy.

Specific objectives:

- Measure the proportion of individuals who complete LTBI treatment (i) among those notified to MSIS with LTBI treatment, and (ii) among those who initiated treatment (at least one dose) by risk-group, age and treatment regimen
- Explore reasons for not completing LTBI treatment among those who initiated treatment (at least one dose), by risk-group, age and treatment regimen
- Assess the risk of severe adverse effects and adverse effects leading to interruption or termination of LTBI treatment among those who initiated treatment (at least one dose), by risk-group, age and treatment regimen
- Explore the feasibility for routine surveillance of LTBI treatment outcome in Norway

Study period: Jan 1, 2016 to Dec 31, 2017

Population and Methods:

- Design: Nationwide prospective cohort
- Period of enrollment: Jan 1, 2016 to Dec 31, 2016
- Population: all cases with LTBI treatment notified to MSIS in the study period
- Sample size: this is determined by the number of cases reported to MSIS with LTBI treatment in the study period. In 2013 and 2014 the number of reported cases was approximately 750 cases a year. This may be lower for the study period as guidelines has focused more on risk groups.

Data: WHO LTBI Taskforce has provided standard indicators for monitoring of LTBI services and this protocol complies with the WHO framework.

We will use demographic and clinical information routinely available through MSIS. Additional data will be collected through a standardized form that will be sent to prescribing clinicians and TB coordinators at the time the case is notified to MSIS

Data will include:

- Demographic information; age, sex, country of origin, time of residence in Norway, occupation, reason for LTBI screening, known exposure to TB (available through MSIS)
- Clinical information; previous TB history, comorbidities (hiv-infection, patients with organ-transplants, end-stage renal disease, cancer, silicosis, diabetes, poor nutrition status or drug abuse), immunosuppressive treatment (steroids, bDMARDs), result of TST, IGRA and Chest X-ray (mostly available through MSIS)
- LTBI treatment: treatment regimen, date of start and end of treatment, whether the treatment was self-administered, given under direct observation (DOT) or a mix, DOT-provider if relevant and whether there was a treatment plan meeting prior to treatment start (some information available through MSIS)
- Treatment completion; treatment completed as defined by relevant clinician
- Reasons for not starting or not completing treatment; patient decision, provider decision, negative IGRA after 8 weeks (in children and susceptible contacts who start LTBI treatment before the final result of TB examination is ready), pregnancy, developed active TB, loss to follow-up, death, moved out of the country, comorbidities or side effects (data not available, will be collected for study purpose)
- Side effects; drug adverse effects leading to interruption or termination of treatment, by risk group and treatment regimen (data not available, will be collected for study purpose)

Numerator: at the end of a full LTBI treatment course, treated individuals are counted among those who completed treatment if adherent to the prescribed regimen as assessed by the prescribing provider, took of at least 80% of the full dose within a period of 150% of the selected duration of therapy, or if number of doses is not available information about treatment outcome classified as completed by the prescribing provider

Denominator: individuals are counted after (i) being notified to MSIS with LTBI treatment, and (ii) taking the first dose of a full treatment course for LTBI.

Adverse effects will be recorded consistent with WHO toxicity grading scale for determining the severity of adverse events and Common Terminology Criteria for Adverse Events, (CTCAE), version 4 (http://evs.nci.nih.gov/ftp1/CTCAE/CTCAE_4.03_2010-06-14_QuickReference_5x7.pdf)

We will send one reminder, before eventually calling the prescribing clinician or responsible TB coordinator.

Analysis: The study is descriptive by nature. Continuous variables will be presented as means and standard deviations for symmetrical data, and as medians and ranges for skewed data. Categorical variables will be presented as frequencies and percentages (%). Associations between pairs of categorical variables will be assessed by Pearson's chi squared or Fisher´s exact tests, as appropriate. The independent samples t-test will be used to explore relationships among continuous variables.

We will stratify analysis by risk groups, age and treatment regimen. We will use logistic regression analyses to explore associations between treatment completions (yes/no) and the following covariates: risk group, age, treatment regimen, origin and comorbidities.

Study partners and institutions: The study is a collaboration between NIPH and collaborating Regional Health Trusts

Funding: Own funding

Ethical considerations: We will seek ethical approval from the Regional Ethical Committee

References

1. WHO. Global strategy and targets for tuberculosis prevention, care and control after 2015. 2013 [cited EB 134/12; Available from: http://apps.who.int/gb/ebwha/pdf_files/EB134/B134_12-en.pdf?ua=1
2. WHO. Guidelines for the management of latent tuberculosis infection. 2014 [cited; Available from: http://www.who.int/tb/publications/ltbi_document_page/en/
3. Stagg HR, Zenner D, Harris RJ, Munoz L, Lipman MC, Abubakar I. Treatment of Latent Tuberculosis Infection: A Network Meta-analysis. Ann Intern Med. 2014 Aug 12.
4. Helse- og omsorgsdepartementet. Forskrift om tuberkulosekontroll FOR-2009-02-13-205; 2009.
5. Arnesen TM, Eide KA, Norheim G, Mengshoel AT, Sandbu S, Winje BA. Tuberkulose i Norge i 2013 med behandlingsresultater for 2012; 2014.
6. Helse- og omsorgsdepartementet. Forskrift om Meldingssystem for smittsomme sykdommer (MSIS-forskriften) FOR-2003-06-20-740. 2003.
7. Olsen AIM, Andersen HE, Amus J, Djupvik JA, Gran G, Skaug K, et al. Management of latent tuberculosis infection in Norway in 2009: a descriptive cross-sectional study. Public Health Action. 2013;3(2).
8. Kunst H, Khan KS. Age-related risk of hepatotoxicity in the treatment of latent tuberculosis infection: a systematic review. International Journal of Tuberculosis & Lung Disease. 2010 Nov;14(11):1374-81.
9. Folkehelseinstituttet, inventor Folkehelseinstituttet, assignee. Forebygging og kontroll av tuberkulose: en veileder Oslo. 2009 2009.
10. Martinson NA, Barnes GL, Moulton LH, Msandiwa R, Hausler H, Ram M, et al. New regimens to prevent tuberculosis in adults with HIV infection. New England Journal of Medicine. 2011 Jul 7;365(1):11-20.
11. Schechter M, Zajdenverg R, Falco G, Barnes GL, Faulhaber JC, Coberly JS, et al. Weekly rifapentine/isoniazid or daily rifampin/pyrazinamide for latent tuberculosis in household contacts. American Journal of Respiratory & Critical Care Medicine. 2006 Apr 15;173(8):922-6.
12. Sterling TR, Villarino ME, Borisov AS, Shang N, Gordin F, Bliven-Sizemore E, et al. Three months of rifapentine and isoniazid for latent tuberculosis infection. New England Journal of Medicine. 2011 Dec 8;365(23):2155-66.

13. Harstad I, Heldal E, Steinshamn SL, Garasen H, Jacobsen GW. Tuberculosis screening and follow-up of asylum seekers in Norway: a cohort study. BMCPublic Health. 2009 2009;9:141.
14. Harstad I, Heldal E, Steinshamn SL, Garasen H, Winje BA, Jacobsen GW. Screening and treatment of latent tuberculosis in a cohort of asylum seekers in Norway. ScandJ Public Health. 2009 11/13/2009.
15. Kan B, Kalin M, Bruchfeld J. Completing treatment for latent tuberculosis: patient background matters. The international journal of tuberculosis and lung disease : the official journal of the International Union against Tuberculosis and Lung Disease. 2013 May;17(5):597-602.
