# Supplementary material for: Treatment completion for latent tuberculosis infection in Norway: a prospective cohort study
Source: BMC Infect Dis. 2018 Nov 19;18:587. doi: 10.1186/s12879-018-3468-z (PMC6245849; doi:10.1186/s12879-018-3468-z)
Supplement: Supplementary file 2 — a, form sent to clinicians for information about treatment completion. b, translation of form sent to clinicians for information about treatment completion. (ZIP 680 kb) [file 12879_2018_3468_MOESM2_ESM.zip › Additional file 2a_formR3.pdf]

Skriv ut

Fjern alt

folkehelseinstituttet

Studie: Resultat av forebyggende tuberkulosebehandling

Studien er godkjent i REK sør-øst (ref 2015/2122). Det er gitt dispensasjon fra taushetsplikten, jf §35 i helseforskningsloven, og fritak fra hovedregelen om å innhente samtykke fra deltagerne. Mer informasjon om studien finnes i følgeskrivet, samt på: http://www.fhi.no/artikler/?id=117384

Punkt 1, 2, 5 og 6 fylles ut for alle, punkt 3 og 4 fylles ut der behandlingsresultatet er ukjent eller behandlingen ikke er gjennomført.

1

Pasientinformasjon

Etternavn ______ Fornavn ______

Fnr ______ evt D-nr ______ evt DUF-nr ______

2

Behandlingsresultat

Er behandlingsregimet endret underveis i behandlingsforløpet? Hvis ja, gi utfyllende kommentar

______

Ble forebyggende behandling fullført (behandlingen gjennomført i samsvar med behandlingsplan)?

Ja ☐ Dato for avsluttning av behandling: ______ → *gå til punkt 5*

Nei ☐ → *gå til punkt 3* *Ukjent* ☐ → *gå til punkt 3*

3

Årsak til ukjent / ikke fullført forebyggende behandling (flere avkrysninger mulig)

☐ Pasienten hentet ikke resept (startet ikke behandling)

☐ Bivirkninger → *gå til punkt 4*

☐ Behandling avsluttet etter pasientens ønske Dato ______

☐ Diagnose avkreftet, behandling avsluttet Dato ______

☐ Pasienten diagnostisert med aktiv tuberkulosesykdom Dato ______

☐ Pasienten døde Dato ______

☐ Behandling avsluttet på grunn av svangerskap Dato ______

☐ Pasienten forsvant fra behandling Dato for siste kontakt ______

☐ Pasienten flyttet Dato ______

☐ Eget ønske ☐ Sendt ut av landet Oppfølging overført til: ______

Annen årsak ______

folkehelseinstituttet

4 *Bivirkninger som var årsak til opphold i/avslutning av behandling*

☐ Behandling *avsluttet* på grunn av bivirkninger Dato ______

☐ *Opphold* i behandling på grunn av bivirkninger

Periode for opphold i behandling fra ______ til ______

Bivirkning (flere avkrysninger mulig)

☐ Levertoksisitet ☐ Utslett m/u kløe ☐ Perifer nevropati ☐ GI-symptomer ☐ Leddsmerter

☐ Tretthet/uvelhet ☐ Søvnløshet ☐ Influensalignende symptomer ☐ Blødninger/blåmerker

Annet ______

Resultat av blodprøver ved tidspunkt for bivirkninger (angi mest unormale prøveresultater)

Dato	ASAT	ALAT	bilirubin	kreatinin	trc

5 *Behandlingsforløpet*

Dato for første konsultasjon ______ Dato for siste konsultasjon ______

Antall konsultasjoner (inkl oppstart) utført av: lege ______ sykepleier ______

Ble det gjort indusert sputum før behandlingsoppstart ☐ Ja ☐ Nei ☐ Ukjent

Ble det gjennomført behandlingsplanmøte ☐ Ja ☐ Nei ☐ Ukjent

Gjennomføringen av behandlingen:

☐ Selvadministrert ☐ Dosett

☐ DOT hele perioden ☐ DOT deler av perioden ☐ Ukjent

Hvis DOT, hvem var ansvarlig: ☐ Hjemmesykepleier ☐ Apotek ☐ Arbeidsplass

☐ Familie Annet ______

6 *Melders navn, adresse og telefonnummer*

Dato:

Utfylt skjema sendes til:
Folkehelseinstituttet
Avdeling for infeksjonsovervåking
Att: Brita Askeland Winje
Postboks 4404, Nydalen
0403 Oslo
