# Supplementary material for: Treatment completion for latent tuberculosis infection in Norway: a prospective cohort study
Source: BMC Infect Dis. 2018 Nov 19;18:587. doi: 10.1186/s12879-018-3468-z (PMC6245849; doi:10.1186/s12879-018-3468-z)
Supplement: Supplementary file 2 — a, form sent to clinicians for information about treatment completion. b, translation of form sent to clinicians for information about treatment completion. (ZIP 680 kb) [file 12879_2018_3468_MOESM2_ESM.zip › Additional file 2b_translationR3.docx]

**Supplementary material 2b (S2). Content of form sent to responsible health care provider for information about LTBI treatment completion**

Please see Appendix 1a for form numbering and outline.

Heading: Study: Outcome of LTBI treatment

The study has been approved by the Regional Committee for Medical and Health Research Ethics (ref 2015/2122). Exemption from confidentiality and exemption from the main rule of obtaining consent from the participants was approved, according to §35 in the Health Research Act. More information about the study is available in the information letter and on the web http://www.fhi.no/artikler/?id=117384

Numbers 1, 2, 5 and 6 should be filled for all participants, numbers 3 and 4 should be filled if treatment completion is unknown or the treatment is not completed.

1. Patient information: surname, given name, government issued identification number or preliminary ID number for immigrants
2. Treatment completion: Has the regimen changed over the course of treatment? If yes, please provide additional comments. Was the LTBI treatment completed according to the individual treatment plan? If yes, date for treatment completion (and go to number 5). If no or unknown, please go to number 3
3. Reasons for unknown or incomplete treatment (several options possible)
   - The patient did not pick up the prescription/did not start treatment
   - Adverse effects (go to number 4)
   - Treatment was terminated based on patient choice, date
   - Diagnosis no longer relevant, treatment stopped, date
   - The patient was diagnosed with TB disease, date
   - The patient died, date
   - Treatment was interrupted due to pregnancy, date
   - The patient was lost to follow-up, date of last contact
   - The patient moved, date
     1. Patients own choice, expelled from the country, follow-up transferred to….
   - Other reasons, please comment:
4. Adverse events as reason for interruption/termination of treatment
   1. The treatment was terminated due to adverse events, date
   2. The treatment was interrupted due to adverse events, date of start and end of interruption in treatment
   3. Adverse events (several options possible)
      1. Hepatotoxicity
      2. Rash with or without itching
      3. Peripheral neuropathy
      4. Gastrointestinal symptoms
      5. Joint pain
      6. Fatigue/nausea
      7. Insomnia
      8. Flu-like symptoms
      9. Hemorrhage/bruises
      10. Other, please comment
   4. Blood test results at time of adverse event (please list the most abnormal test results)
      1. Date, ASAT, ALAT, bilirubin, creatinine and platelets
5. The course of treatment
   1. Date of first consultation, date of last consultation
   2. Number of consultations (including the consultation when the treatment was decided) provided by physician and by nurse
   3. Was induced sputum taken before treatment initiation? yes/no/unknown
   4. Was there a treatment plan meeting prior to treatment initiation?
   5. Patient support and drug administration throughout treatment:
      1. Self administered
      2. Dosette boxes (pill box)
      3. Unknown
      4. Direct observed treatment (DOT) for part of the treatment period. If yes, who was the DOT provider:
         1. Home visiting nurse
         2. Pharmacy
         3. Workplace
         4. Family
         5. Other, please comment
      5. DOT throughout the treatment period. If yes, who was the DOT provider:
         1. Home visiting nurse
         2. Pharmacy
         3. Workplace
         4. Family
         5. Other, please comment
6. Contact information (name, institutional address and telephone number) of the health care provider who completes the form
