# Supplementary material for: Treatment completion for latent tuberculosis infection in Norway: a prospective cohort study
Source: BMC Infect Dis. 2018 Nov 19;18:587. doi: 10.1186/s12879-018-3468-z (PMC6245849; doi:10.1186/s12879-018-3468-z)
Supplement: Supplementary file 3 — a sensitivity analysis exploring the associations between treatment non-completion, and treatment support, origin, age and sex after imputation for missing values. (PDF 472 kb) [file 12879_2018_3468_MOESM3_ESM.pdf]

Supplementary material, Additional file 3.

Associations between treatment non-completion, treatment support, origin, age and sex after imputation for missing values (n=726)

Covariates	Univariable		Multivariable			
	cOR	P	aOR	SE	P	95% CI
**Origin**						
Foreign-born	1 (ref)		1 (ref)			
Norwegian-born	2.1	0.015	0.8	0.358	0.667	0.36 – 1.94
**Age**						
< 35	1 (ref)		1 (ref)			
> 35 years	1.9	0.023	1.5	0.464	0.176	0.83 – 2.76
**Sex**						
Female	1 (ref)		1 (ref)			
Male	0.5	0.017	0.6	0.165	0.063	0.35 – 1.03
**Treatment support.** Model with interaction term for origin						
Effect of treatment support on treatment completion in foreign-born						
Self administered [I]	1 (ref)		1 (ref)			
DOT daily [II]	0.2	0.004	0.3	0.139	0.011	0.10 – 0.74
DOT weekly [III]	0.4	0.012	0.5	0.167	0.033	0.22 – 0.94
Effect of treatment support on treatment completion in Norwegian-born						
Self administered [I]	1 (ref)		1 (ref)			
DOT daily [II]	2.1	0.231	2.5	1.618	0.165	0.68 – 9.04
DOT weekly [III]	0.9	0.877	1.0	0.806	0.968	0.19 – 4.96

OR: Odds ratio, SE: standard error, DOT: Direct Observed Treatment for part of or the full treatment period

[I] *Self-administered include those who managed their treatment themselves or were given weekly pill boxes*

[II] *daily DOT include those who were administered daily treatment under direct observation*

[III] *weekly DOT include those who were administered weekly rifapentine and isoniazid under direct observation*
